# Assessing burnout among Obstetrics & Gynecology residents during night float versus day float in a large academic hospital

**DOI:** 10.1186/s12909-022-03897-4

**Published:** 2022-11-24

**Authors:** Miriam Tarrash, David Nelson, Nagaraj Gabbur, Gary L. Goldberg

**Affiliations:** 1 Department of Obstetrics & Gynecology, Zucker School of Medicine at Hofstra/Northwell and Northwell Health, New York, USA; 2 Department of Obstetrics & Gynecology LIJ Medical Center, Ob/Gyn Admin Suite Room C221, 270-05 76th Avenue, New Hyde Park, New York, NY 11040 USA

**Keywords:** Burnout, Residents, Obstetrics & Gynecology

## Abstract

**Background:**

The prevalence estimates of burnout among residents vary widely. Resident physicians working overnight have additional stressors and therefore, may be at higher risk of developing burnout.

**Objective:**

To determine the rates of burnout among residents working night rotations versus day rotations.

**Methods:**

This is a prospective, cross sectional, survey-based assessment of the prevalence of burnout among Obstetrics and Gynecology (OBGYN) residents on nights versus days rotations conducted at a large academic residency program that spans two separate hospitals in New York. All residents in the residency program were asked to complete the Maslach Burnout Inventory – Human Services Survey for Medical Personnel (MBI-HSS (MP)) after the first rotation of the academic year in 2018, 2019, and 2020. The results for each of the three aspects of the MBI-HSS (MP): emotional exhaustion, depersonalization, and personal accomplishment, were then compared for those on nights versus day rotations using students t-test.

**Results:**

A total of 76 responses were received, 13 from residents on night rotations and 63 from residents on day rotations with a response rate of 61.8%. Comparing resident responses for a night versus day rotation, the residents averaged a low level of emotional exhaustion (a score of 17 ± 9) on day shift, compared to a moderate level of emotional exhaustion (a score of 18 ± 14) on nights (*p* = 0.37). Similarly, 55.6% of respondents reports low personal accomplishment on days, compared to 76.9% while on nights.

**Conclusions:**

Emotional exhaustion scores were lower for residents on daytime rotations (mean score 17, SD 9), compared to those on nights rotations (mean 18, SD 14). Although there was no difference in depersonalization when comparing the day and night shift, 45% of the responses indicated high levels of depersonalization regardless of the type of shift. These results highlight the need to continue efforts to minimize burnout in medical training.

## Introduction

Burnout is defined as an emotional depletion and loss of motivation from prolonged exposure to chronic workplace stress that has not been successfully managed. Three dimensions have been used to describe the complex syndrome of burnout: emotional exhaustion, depersonalization, and feelings of decreased personal accomplishment [1, 2]. Studies show that more than half of all US physicians experience professional burnout, and the incidence is increasing [3]. Burnout is increasing in prevalence with 45.5% of physicians surveyed in 2011 reporting symptoms of burnout compared to 54.4% in 2018. Additionally, feelings of an adequate work-life balance are declining with 48.5% in 2011 and 40.9% in 2014 reporting an inadequate experience [4]. Physicians have been shown to have higher levels of burnout, when compared with other professions. The negative impacts of physician burnout extend across multiple levels in healthcare putting the hospital system, physicians themselves and their patients at risk of unsafe events. Cognitive impairment, including reduced executive function, appears to be one burnout consequence which could have detrimental effects on patient safety [5]. Not only are professional consequences of burnout evident, but also, personal sequela are possible too including substance abuse disorders and suicide [6].

Those who are completing residency training are exposed to unique factors that may contribute to elevated rates of burnout. After completion of Medical School, residency training is a requirement for physicians. It requires a competitive resume, higher education, taxing work hours, relatively restricted income, and lack of autonomy. As a result, resident physicians’ mental health may be impacted and may increase the likelihood of burnout in this population. The prevalence estimates of burnout among residents vary widely between medical and surgical specialties, gender, geographic location, and post-graduate year in training, therefore, the exact prevalence of burnout remains unknown [7, 8]. Systemic causes of burnout involve issues in the learning environment and overall institutional culture [9, 10]. Workplace climate and the hierarchy in medicine clearly has an influence on overall resident well-being. Notoriously, residents in surgical specialties, those in their intern year, and those who identify as female have had a higher rate of burnout [11, 12]. Previous studies have also suggested that personality traits such as pessimism, neuroticism and high conscientiousness and those self-identifying as anxious or disorganized are more likely to experience burnout [13]. However, emotional intelligence and perseverance are strong predictors of resident well-being [14–16]. Residents experiencing symptoms of burnout are at an increased risk of delivering suboptimal patient care and contributing to an increase in medical errors [4, 16–18]. Residents are also at a higher risk of substance use, alcohol consumption, depression, and suicidal thoughts and attempts [6, 11, 19–21].

Despite increased focus on improving residency, limited information is known about risks for burnout in this population. Residency programs and program directors have a responsibility to both trainees and patients to facilitate an experience with reduced burnout. There are various factors that may affect perceived burnout that are different during day shift hours when compared with night shift hours, includes fewer support staff, fewer social interactions outside of the workplace, and difficulty adjusting to an opposite sleep schedule. The goal of this study was to determine the rates of burnout among residents working night rotations versus day rotations.

## Methods

This is a prospective, cross sectional, survey-based assessment of the prevalence of burnout among Obstetrics and Gynecology (OBGYN) residents on nights versus days rotations conducted at a large academic residency program that spans two separate hospitals in New York. The residents and attendings cover an extremely high-volume and high acuity labor and delivery (L&D) floors with approximately 17,000 deliveries per year. The L&D are accompanied by a level 3 and a level 4 NICU, so many transferred for neonatal concerns are received. The residents in all post-graduate years in a large OB/GYN residency program were asked to complete the Maslach Burnout Inventory – Human Services Survey for Medical Personnel (MBI-HSS (MP)) and indicate which rotation they just completed after the first rotation of the academic year in 2018 and again in 2019 and 2020.

The MBI-HSS (MP) is a validated tool and is considered the gold standard for evaluating burnout in medical professionals [22]. The MBI-HSS (MP) assesses and reports a numeric score in each of three domains of emotion exhaustion (range 0 to 42), depersonalization (range 0 to 42), and personal achievement (range 0 to 48). Outcome measures were quantified as the mean scores in each of the three domains.

The questionnaire was distributed via the Research Electronic Data Capture (REDCap). Those filling out the questionnaire were asked to respond to each question indicating the frequency with which he or she experiences the sentiment of each statement. These statements are intended to evaluate the degree of satisfaction and pride the resident feels regarding how they perform their job. The respondents were blinded from their scores and from the thresholds for low, moderate, and high values in each section, to help prevent the respondent from answering based on his or her own subjective level for each category.

For each resident, we derived scores for each of the three domains of emotional exhaustion, depersonalization, and personal accomplishment. We then divided responses based on the type of rotation and calculated mean scores for residents on night rotation or daytime rotations. We assessed for differences in mean scores between day and night rotations in the domain’s categories via students t-test. Consistent with prior work, we also applied cut-off scores based on previously published normative data [22] to divide responses into low, moderate, and high score groups in each of the three domains [23–26]. Emotional exhaustion scores were divided into low (0–17 points), moderate (18–29 points), and high (30–42 points) score groups and depersonalization was divided into low (0–5), moderate (6–11), and high (12–42) score groups. Given its inverse relationship with burnout, personal achievement scores were grouped into low (40–48), moderate (34–39), and high (0–33) groups. The review and analysis of data collected was approved by the Northwell Institutional Review Board.

## Results

A total of 76 responses were received, 13 of which were from residents on night rotations and 63 from residents on day rotations. Each year 41 residents were surveyed from PGY-1, -2, -3 and -4 leading to a maximum number of responses of 123, yielding a response rate of 61.8% (Table 1).

**Table 1:** Respondents by Level of Training

Level of Training	Day rotation n	Night rotation n	Total n (%)
PGY-1	14	3	17 (22.4)
PGY-2	16	3	19 (25.0)
PGY-3	17	2	19 (25.0)
PGY-4	16	5	21 (27.6)

Comparing resident responses for a night versus day rotation, the residents averaged a low level of emotional exhaustion (a score of 17 ± 9) on day shift, compared to a moderate level of emotional exhaustion (a score of 18 ± 14) on nights (*p* = 0.37). Additionally, the residents averaged a moderate level of personal accomplishment (a score of 39 ± 10) on day shift, compared to a low level of personal accomplishment (a score of 41 ± 14) on nights (*p* = 0.17). Though there was no difference in depersonalization when comparing day and night shift (scores of 14 ± 7 and 16 ± 7 respectively; *p* = 0.21), 45% of the responses indicated high levels of depersonalization (Table 2).

**Table 2:** MBI-HSS (MP) results for residents on day versus night rotations

	**Day rotation** **(Mean, SD)**	**Night rotation** **(Mean, SD)**	***P*-value**
Emotional Exhaustion	17 ± 9	18 ± 14	0.37
Depersonalization	14 ± 7	16 ± 7	0.21
Personal Accomplishment	39 ± 10	41 ± 14	0.17

Emotional exhaustion score: low (0–17), moderate (18–29), and high (30–42)

Depersonalization score: low (0–5), moderate (6–11), and high (12–42)

Personal achievement score: low (40–48), moderate (34–39), and high (0–33)

**Fig. 1:**
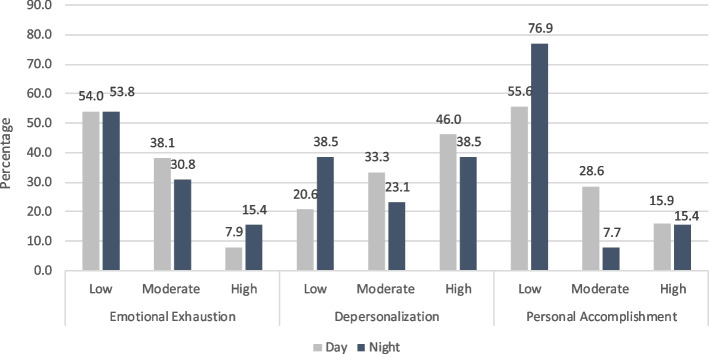
Percentage of results from three domains for residents on day versus night rotations

Percent responses were calculated from each domain as displayed in Fig. 1. Notably, 55.6% of respondents reported low personal accomplishment on days, compared to 76.9% while on nights.

## Discussion

This study found higher measures of burnout in residents on night shifts versus day shifts. These findings are in line with studies that report decreased quantity of sleep and reduced cognition on night floats [27, 28]. However, no study to date has specifically analyzed the differences in resident burnout between night and day shifts. These results show moderate emotional exhaustion and low personal accomplishment while on nights compared to low emotional exhaustion and moderate personal accomplishment on day rotations. This further reinforces the difficulties that residents experience while on night rotations for these two domains. Residents on both day and night rotations experience high rates of depersonalization, highlighting the need for improvements in medical training.

Our data reveals higher rates of self-reported burnout for residents on night shifts when compared to days. Many factors that may affect perceived burnout which are exacerbated at night include fewer support staff, fewer social interactions outside of the workplace, and difficulty adjusting to an opposite sleep schedule. Working overnight (night shift) has been associated with adverse effects such as sleep deprivation, irritable mood, reduced quality of patient care and diminished healthcare worker personal safety. Studies have concluded that residents are at risk when driving home after a night on call emphasizing the importance of post-call recovery sleep and the consequences of sleep disruption and deprivation. Returning to a baseline sleep schedule after a block of night call is extremely difficult [27]. Additionally, there is an increased propensity to ignore healthy nutrition and perform less physical activity. The alteration in circadian rhythm increases the risk of numerous medical conditions and malignancy [29, 30]. On a night shift, the junior resident has limitations in easy access to senior residents and faculty. This is of upmost importance in their education, development and clinical training [31].

Physicians are strongly predisposed to burnout due to intense clinical demands in work hours, continued education, large personal debt, and sacrifices in personal relationships. Imposter syndrome, depersonalization and a lower level of training have a tremendous impact on overall resident stress [32]. Residents are predisposed to depression and those experiencing symptoms of burnout are at an increased risk of delivering suboptimal patient care and contributing to an increase in medical errors [17, 19, 20]. These factors highlight the need for continued research in order to effectively analyze the impacts of chronic occupational stress and to better quantify the amount of people impacted by the phenomenon of burnout.

Efforts to ensure protections for residents have been aimed at restricting work hours. The enactment of Accreditation Council for Graduate Medical Education (ACGME) work hour limits stating that residents should work no more than 80 h per week were associated with an improvement in emotional exhaustion and burnout [33]. Patient mortality seemed to improve in addition to resident well-being [34]. One conflicting study found that the reduction in resident work hours from an average of 100 to 82 following resident duty hour restrictions did not significantly change measures of burnout [35]. Limiting the number of on-call hours is essential to promote a healthy work-life balance, maintain necessary sleep habits and attempt to alleviate and prevent burnout. Additionally, studies have shown that the implementation of a night float system was associated with decreased sleep hours, while 24-h coverage led to improved junior case volume and elective time with no detrimental effect on patient-related outcomes [28, 36, 37]. Since trainee burnout has been associated with both night shifts and medical errors, focused efforts aimed at minimizing or improving night shift work during medical training are necessary. Additional studies are necessary to determine the benefits and outcomes when comparing a night-float schedule to a 24-h call schedule [38]. Our data suggests that residents appear to be more prone to experience emotional exhaustion while working any night shifts. Strategies to combat this should be considered by residency programs including extra support (mid-level clinicians), limiting, or removing night rotations while balancing the need for adequate training.

The Covid-19 pandemic occurred during the course of our study and created unique healthcare challenges that had a major impact across all specialties. Hospital systems that were overwhelmed with patients required residents to be responsible for a higher number of patients and work hours while battling the substantial work and life stressors of their own personal illness prevention [39, 40]. This resulted in a spike in the prevalence of resident burnout, anxiety, distress, and isolation [41, 42]. Studies throughout the course of the Covid-19 pandemic highlight the concerns of residents including anxiety about their professional future due to the perception that their training suffered a significant and irreversible impairment [43]. Furthermore, adverse effects spread beyond the workplace into all aspects of their personal and social lives [44]. Extra-ordinary hardships have been magnified during this time for all health care clinicians.

The MBI-HSS (MP) has unique strengths and weaknesses. This is a validated survey tool which relies on previously published data derived from metagroups of respondents creating standardized metrics [22]. It is widely used and considered the gold standard for assessing burnout in medical professionals [22]. One limitation of this study includes differences in MBI-HSS (MP) cutoff scores to define burnout among studies [23–26]. However, for our analysis, the latest edition of the Maslach Burnout Inventory Manual (Fourth Edition) was used. Additional limitations of this tool includes the variability in the prevalence of burnout, distinct cutoff values, and the self-reported nature of the survey. Although the average scores for emotional exhaustion and personal accomplishment in our study fell into different sections (low, moderate, and high), the numeric values were not statistically significant because our study was limited by a small sample size.

Another limitation in this area of research is that burnout rates are highly variable. In reality, burnout exists on a continuum rather than discrete categories and is subject to fluctuate over time. This study had a relatively small sample size and future studies would benefit from conducting a survey across multiple institutions. Other considerations of the study include temporal factors such as the Covid-19 pandemic. Some of the surveys were taken prior to COVID while others were taken during the pandemic. There is a high likelihood that resident responses were skewed by the stresses of COVID. Additionally, to control for different stressors during different points in the residency year such as seasonal variations, all residents were surveyed at the same time. While this should have controlled for any temporal confounding variables, it ultimately compared different residents who were on night shift than those on day shift. Another possible method of controlling for confounding variables between different residents could have been to survey each resident during his or her night rotation and again during a day rotation. However, this would have resulted in residents being surveyed at different times during the year which would not have controlled for temporal variables.

Resident well-being is of the upmost importance and residents are unlikely to seek help on their own [45]. There is a shared responsibility of both healthcare systems and individuals to promote a rich learning environment which minimizes the risks of burnout. Implementation of formal programs focused on resident well-being have positively impacted residents’ perceived stress, life satisfaction and their perception of the residency program [46, 47]. Organizational support by program directors and other faculty in leadership positions can help promote well-being [48]. Enacting workshops, residency assistance programs, self-care interventions, engagement, support groups, didactic sessions, stress management and coping training are all effective strategies to build resilience and feelings of social belonging [49–51]. Efforts should be focused on improving access to mental health clinicians for medical residents [52]. Additionally, healthy exercise habits have been associated with a lower risk of burnout and higher quality of life among medical trainees [53]. Although many gaps in knowledge about preventing burnout remain, strategies to enhance medical training and search for effective cures for burnout must continue.

## Conclusions

Emotional exhaustion scores were lower for residents on daytime rotations (mean score 17, SD 9), compared to those on nights rotations (mean 18, SD 14). Though there was no difference in depersonalization when comparing day and night shift 45% of the responses indicated high levels of depersonalization regardless of the type of shift. These results enhance our understanding of this topic and highlight the need to continue efforts to minimize burnout in medical training, especially for those working nights.

## Data Availability

All data generated or analyzed during this study are included in this published article.
